# Low cerebrospinal fluid pressure predisposes to postoperative epidural hematoma following lumbar decompression: a potentially modifiable physiological risk factor

**DOI:** 10.1186/s12891-026-09836-4

**Published:** 2026-04-15

**Authors:** Hiromi Kumamaru, Takeyuki Saito, Shingo Yoshizaki, Toshiki Konishi, Yasuharu Nakashima, Katsumi Harimaya

**Affiliations:** 1 Department of Orthopaedic Surgery, Kyushu University Beppu Hospital, 4546 Tsurumibaru, Beppu, Oita Japan; 2 Department of Orthopaedic Surgery, Graduate School of Medical Sciences, Kyushu University, Fukuoka, Japan

**Keywords:** Cerebrospinal fluid pressure, Spinal epidural hematoma, Lumbar decompression surgery, Risk factors, Perioperative management.

## Abstract

**Background:**

Postoperative spinal epidural hematoma (SEH) is a serious complication of lumbar spinal surgery. While various risk factors have been reported, the role of cerebrospinal fluid (CSF) pressure in SEH development remains unclear. This study aimed to investigate the association between preoperative CSF pressure and postoperative SEH, and to explore potentially modifiable physiological factors.

**Methods:**

A total of 94 patients undergoing lumbar decompression surgery were retrospectively reviewed. Preoperative CSF pressure was measured via lumbar puncture in the lateral position before surgery. Patients were categorized into hematoma and no-hematoma groups based on postoperative magnetic resonance imaging findings. Clinical and surgical variables were compared between groups, and multivariable logistic regression analysis was performed to identify independent risk factors for SEH.

**Results:**

Postoperative epidural hematoma with significant dural sac compression was observed in 30.8% of patients. Multivariable logistic regression analysis revealed that lower CSF pressure (adjusted OR = 0.87 per 10 mmH_2_O increase; 95% CI: [0.74–0.98], *P* = 0.028) and the number of decompressed levels (adjusted OR = 1.94; 95% CI: [1.15–3.47], *P* = 0.017) were independently associated with SEH development. Post hoc power analysis for CSF pressure yielded sufficient power of 0.81.

**Conclusions:**

Low preoperative CSF pressure was independently associated with postoperative SEH and may represent a potentially modifiable physiological factor. Perioperative strategies aimed at maintaining adequate CSF pressure may help reduce the risk of postoperative epidural hematoma, although their clinical effectiveness requires confirmation in prospective studies.

## Introduction

Postoperative spinal epidural hematoma (SEH) is a potentially devastating complication of lumbar spine surgery, causing acute neurological deterioration and compromising both functional and radiological outcomes [1, 2]. Symptomatic SEH after lumbar surgery is rare, occurring in 0.1–0.3% of cases, with only 32 emergency surgical interventions reported among 14,942 procedures [3–9]. In contrast, small hematomas that cause only mild symptoms—such as minor leg or back pain without neurological deficits—are considerably more common, with an incidence reported between 33% and 67% following lumbar spine surgery [2, 10–12]. Although often asymptomatic or minimally symptomatic, these postoperative SEHs have been shown to contribute to radicular symptoms, delayed mobilization, or prolonged functional recovery [13]. Their high frequency has led to increased interest in the diagnosis and underlying mechanisms of such hematomas [2, 13].

Several risk factors for postoperative epidural hematoma have been identified, including advanced age, female sex, multilevel procedures, revision surgery, perioperative hypertension, intraoperative blood loss, coagulation abnormalities, and intraoperative use of Gelfoam for dural coverage [3–8]. Previous studies have predominantly focused on hemorrhagic or coagulation-related mechanisms as risk factors for SEH. However, some reported factors—such as age, sex, or the use of Gelfoam—are unlikely to directly affect coagulation, implying that additional physiological or hemodynamic processes may be involved.

One potential mechanism involves cerebrospinal fluid (CSF) pressure. Theoretically, reduced CSF pressure could lead to epidural venous engorgement or congestion, producing a hemodynamic environment that favors hematoma formation [14–16]. This hypothesis is supported by magnetic resonance imaging (MRI) findings showing epidural venous dilation in patients with CSF hypotension following lumbar puncture or spontaneous intracranial hypotension [15, 17]. Despite these insights, no clinical study has directly investigated whether low CSF pressure serves as an independent clinical risk factor of postoperative SEH. Therefore, this study aimed to examine whether preoperative CSF pressure is associated with the development of postoperative SEH in patients undergoing lumbar decompression surgery, to clarify a previously unaddressed aspect of SEH pathophysiology and its potential relevance to perioperative management.

## Methods

This study was approved by the Kyushu University Institutional Review Board (IRB approval No. 29–411). Inclusion criteria involved patients who underwent lumbar decompression surgery without fusion at our institution between April 2021 and April 2022. Exclusion criteria included patients with trauma, tumor, or infection, those undergoing simultaneous fusion surgery, and those treated with microendoscopic decompression, due to differences in surgical invasiveness and postoperative epidural space conditions that may affect hematoma formation and MRI evaluation. After applying these criteria, a total of 94 patients who underwent conventional open lumbar decompression surgery performed by four board-certified spine surgeons at our institution (90 laminotomies and 4 laminectomies). The preoperative diagnoses included spinal stenosis, degenerative spondylolisthesis, and lumbar disc herniation. A 15-Fr drain was placed after surgery and removed within 2 days. Postoperative care involved maintaining a neutral spine position by keeping patients in the supine or lateral position on the day of surgery. Patients were allowed to sit and ambulate beginning on postoperative day 1.

Preoperative CSF pressure was measured during diagnostic lumbar puncture performed as part of preoperative myelography within 2 months prior to surgery, with patients in the lateral decubitus position and relaxed, without sedation. The table was maintained horizontal, and the measurement level was referenced to the needle hub. Pressure was recorded in mmH_2_O once the column stabilized.

Potential risk factors in this study were selected based on previously published reports suggesting their relevance to postoperative SEH [2–13]. Given recent evidence demonstrating an association between frailty and postoperative complications, the modified frailty index was also included in the analysis [18, 19]. Comprehensive review of each patient’s medical records was performed.

Preoperative clinical data were obtained from standard patient intake forms and included age, sex, body mass index (BMI), smoking status, daily alcohol consumption, and the American Society of Anesthesiologists Physical Status (ASA-PS). Frailty was assessed using the modified 5-item frailty index (mFI-5) [18, 19], which includes five preoperative variables: congestive heart failure within 30 days, diabetes mellitus, chronic obstructive pulmonary disease or pneumonia, hypertension requiring medication, and partially or completely dependent functional status. One point was assigned for each variable present, yielding a total frailty score ranging from 0 to 5. Patients were categorized into frail (mFI-5 ≥ 2) and non-frail (mFI-5 ≤ 1) groups based on previously validated cut-off values used in spinal surgery studies [18, 19]. Systolic and diastolic blood pressure (SBP and DBP) were recorded upon hospital admission. Laboratory evaluations performed within 8 weeks prior to surgery included platelet count, prothrombin time-international normalized ratio (PT-INR), and activated partial thromboplastin time (APTT).

In patients receiving anticoagulant or antiplatelet therapy, aspirin was continued throughout the perioperative period. Clopidogrel was discontinued 7 days before surgery and resumed 3 days postoperatively. Direct oral anticoagulants (e.g., apixaban, rivaroxaban, edoxaban, dabigatran) were routinely stopped 2 days before surgery and restarted 3 days after.

Intraoperative data included the number of decompressed levels, total operative time, whether discectomy was performed, occurrence of intraoperative durotomy, revision surgery, and whether L2–3 decompression was conducted. Postoperative data included total drain output, maximum systolic and diastolic blood pressure (SBP and DBP) immediately after extubation, and average SBP and DBP within the first 3 h postoperatively.

Preoperative MRI was performed within 2 months before surgery, and postoperative MRI was obtained at a median of 2 days after surgery (range, 1–5 days) in all patients, which corresponds to the early postoperative period when SEH size is unlikely to show substantial radiological change. Three board-certified spine surgeons (H.K., T.S., and S.Y.) with 8 to 19 years of clinical experience evaluated the images in a blinded manner. Given the limited accuracy of hematoma volume quantification on early postoperative MRI [20, 21], we used the dural sac cross-sectional area (CSA) as a surrogate marker for SEH severity. CSA measurement serves as a reproducible measure for evaluating postoperative epidural hematoma severity [1, 10, 13]. CSA was measured manually on T2-weighted axial images at each lumbar disc level (L1/2 to L5/S1) using the area measurement tool in the Picture Archiving and Communication System (PACS; Fujifilm, Japan). All measurements were performed independently by the three raters, and their values were averaged. Interobserver reliability for CSA was supported by a Cronbach’s alpha of 0.96. Postoperative CSA was assessed similarly to evaluate dural sac compression, and the average of the three measurements was used. Interobserver reliability for postoperative CSA was 0.95. The expansion ratio of the CSA was calculated by dividing the postoperative by the preoperative CSA at each disc level.

**Fig. 1:**
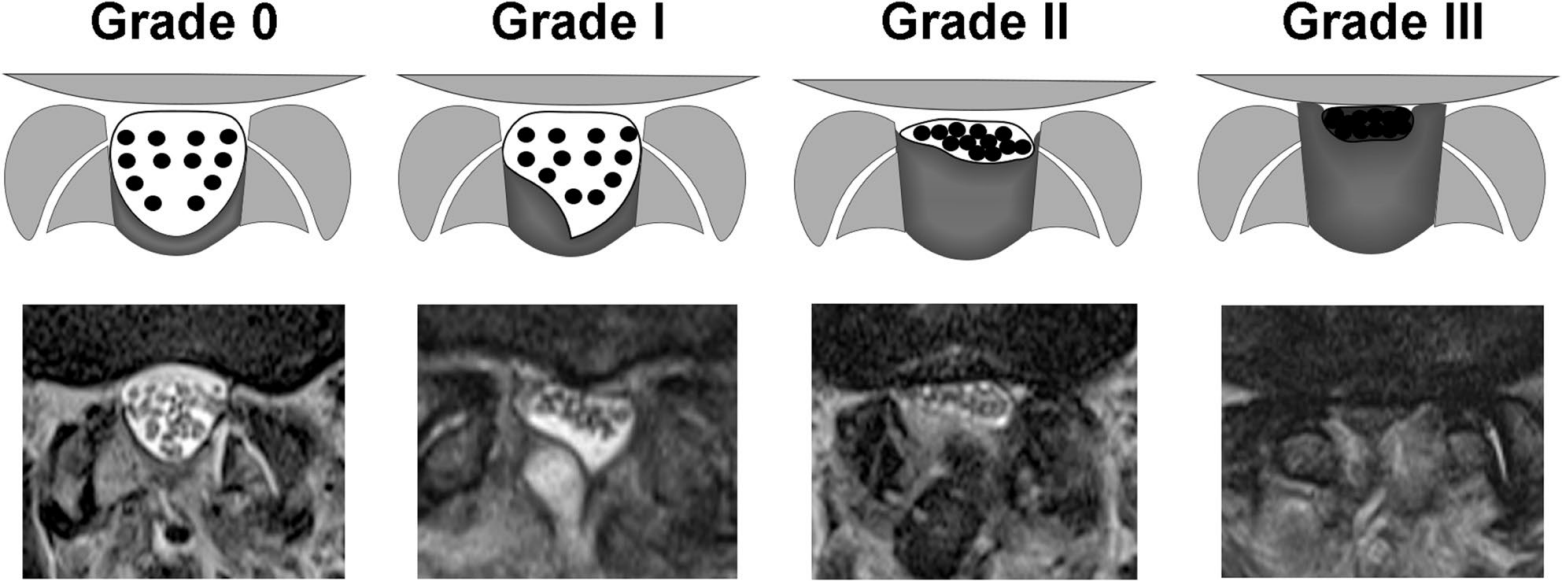
Classification of postoperative spinal epidural hematoma on axial T2-weighted MRI. Grade 0 – No hematoma. The dural sac appears round with clearly visible CSF. Grade I – Mild dural sac compression. Nerve roots are separated and identifiable, with preserved CSF signal. Grade II – Moderate compression. Nerve roots are crowded but still individually recognizable, with partial CSF preservation. Grade III – Severe compression. CSF is absent and nerve roots appear indistinct or obscured

Postoperative SEH was classified into four grades based on axial T2-weighted MR images (Fig. 1): grade 0 (no hematoma, dural sac appears round with clearly visible CSF), grade I (mild dural sac compression; nerve roots are separated and identifiable, with preserved CSF signal), grade II (moderate compression; nerve roots are crowded but still individually recognizable, with partial CSF preservation), and grade III (severe compression; CSF is no longer visible, and nerve roots appear indistinct or obscured). Three board-certified spine surgeons independently assessed SEH at each lumbar level, with a kappa coefficient of 0.82 for interobserver agreement. One-way ANOVA with post hoc Tukey HSD was used for grade comparisons. Postoperative SEH was defined as MRI grade II or III, irrespective of symptoms, to include subclinical but physiologically meaningful hematomas as previously described [13]. Patients with grade II or III hematoma in at least one spinal level were categorized into the hematoma group; the remainder comprised the no-hematoma group. In the hematoma group, the smallest postoperative dural sac CSA was used for analysis.

Univariate analyses were performed to identify potential risk factors for SEH. Variables with *P* < 0.1 were considered for inclusion in the multivariable logistic regression model. Because age and DBP are known physiological determinants of CSF pressure [22, 23], their simultaneous inclusion could lead to overadjustment. Therefore, these variables were excluded from the primary model. Instead, preoperative PT-INR [2] and anticoagulant therapy [6, 24, 25], both previously reported as clinically relevant risk factors, were included irrespective of their univariate significance. Sensitivity analyses were performed by adding age and/or DBP to the model to examine the stability of the association between CSF pressure and postoperative SEH. Continuous variables were expressed as mean ± standard deviation (SD) and compared between groups using the Student’s t-test or Mann–Whitney U test. Model assumptions were evaluated as follows. The linearity of the logit for continuous predictors was assessed using the Box–Tidwell approach. Multicollinearity was examined using variance inflation factors (VIF) derived from a corresponding linear model including all explanatory variables; VIF < 5 was considered acceptable. Model discrimination was quantified by the area under the receiver-operating-characteristic curve (AUC) based on predicted probabilities. Categorical variables were compared using the chi-square test or Fisher’s exact test as appropriate. All analyses were conducted using JMP Pro 15 (SAS Institute, Cary, NC, USA). A two-tailed P value of < 0.05 was considered statistically significant.

## Results

This study included 94 patients, 29 women and 65 men, with a mean age of 71.7 years (24–88 years). The SEH grading results are summarized in Table 1. Of the 210 segments, 129, 43, 17, and 21 were categorized as grades 0, I, II, and III, respectively. The mean dural sac CSA for grades 0, I, II, and III were 136.8 ± 34.7 mm^2^, 109.7 ± 32.9 mm^2^, 82.1 ± 21.8 mm^2^, and 57.6 ± 20.1 mm^2^, respectively. Significant differences were observed in the dural sac CSA among all groups (One-way ANOVA with post hoc Tukey HSD. Grade I vs. II, *P* = 0.0273; Grade II vs. III, *P* = 0.0388; other comparisons, *P* < 0.0001), indicating the reliability and validity of the SEH analysis.

**Table 1:** Dural sac CSA by hematoma grade

Variable	Grade 0	Grade I	Grade II	Grade III
Number of segments	129	43	17	21
Dural sac CSA (mm²)	136.8 ± 34.7	109.7 ± 32.9	82.1 ± 21.8	57.6 ± 20.1

Values are expressed as mean ± SD

*CSA* Cross-sectional area

Of the 94 patients, 29 patients (30.8%) presenting with grade II or III hematomas at any segment were classified into the hematoma group. In contrast, the remaining 65 (69.2%) were categorized into the no-hematoma group. Preoperative dural sac CSA was 79.3 ± 32.2 mm^2^ and 68.9 ± 31.6 mm^2^ in hematoma and no-hematoma groups, respectively (*P* > 0.05) (Table 2). Postoperative dural sac CSA was significantly smaller in the hematoma group (67.9 ± 24.4 mm²) than in the no-hematoma group (131.1 ± 35.5 mm², *P* < 0.0001). Significant differences were observed in the expansion ratio of CSA between the two groups (99.5 ± 52.4% in the hematoma group vs. 222.9 ± 112.1% in the no-hematoma group; *P* < 0.0001). No patient exhibited progressive neurological deficits or required emergency reoperation due to postoperative epidural hematoma. However, postoperative radicular pain was more frequently observed in the hematoma group (11 patients, 37.9%) compared to the no-hematoma group (8 patients, 12.3%; *P* = 0.0134). Four (13.8%) patients in the hematoma group exhibited cephalocaudal extensions beyond the decompression levels.

**Table 2:** Comparison of dural sac CSA and postoperative symptoms between groups

Variable	Hematoma group (*n* = 29)	No-hematoma group (*n* = 65)	*P* value
Preoperative dural sac CSA (mm²)	79.3 ± 32.2	68.9 ± 31.6	0.118
Postoperative dural sac CSA (mm²)	67.9 ± 24.4	131.1 ± 35.5	< 0.0001*
Expansion ratio of CSA (%)^a^	99.5 ± 52.4	222.9 ± 112.1	< 0.0001*
Postoperative radicular pain, n (%)^b^	11 (37.9%)	8 (12.3%)	0.013*

Values are expressed as mean ± SD or number (%)

*CSA* Cross-sectional area

^a^Expansion ratio = (postoperative CSA / preoperative CSA) × 100

^b^Defined as newly developed or worsened leg pain after surgery

^*^P < 0.05, considered statistically significant

The univariate analysis identified potential risk factors for postoperative SEH, including age (*P* = 0.039), preoperative CSF pressure (*P* = 0.031), number of decompressed levels (*P* = 0.020), and maximum DBP (*P* = 0.022) (Tables 3 and 4). The frailty index was not significantly associated with SEH. In the subsequent multivariable logistic regression analysis, age and maximum DBP were excluded due to their known influence on CSF pressure and potential confounding effects [22, 23]. Instead, preoperative PT-INR and anticoagulant therapy—variables previously reported as clinically relevant risk factors—were included. The Box–Tidwell tests showed no violation of the logit linearity for continuous predictors (all *P* > 0.05). All VIF values were below 2.0 (range, 1.03–1.06), indicating no significant collinearity among covariates. The model satisfied key assumptions and achieved moderate discrimination (AUC = 0.71). The analysis revealed that lower preoperative CSF pressure (adjusted OR = 0.87 per 10 mmH_2_O increase; 95% CI: [0.74–0.98]; *P* = 0.028) and a greater number of decompressed levels (adjusted OR = 1.94; 95% CI: [1.15–3.47]; *P* = 0.017) were independently associated with postoperative SEH (Table 5). When maximum DBP was added to the model, this association persisted (adjusted OR = 0.88 per 10 mmH_2_O increase; 95% CI: [0.78–0.99]; *P* = 0.036). However, when age was included—either alone (*P* = 0.877) or together with DBP (*P* = 0.928)—the association between CSF pressure and SEH was attenuated and became non-significant, suggesting that age shares physiological variance with CSF pressure. This attenuation likely reflects shared physiological variance, as age is inversely correlated with CSF pressure. Overall, the results indicate that lower preoperative CSF pressure substantially contributes to postoperative SEH risk. Each 10 mmH_2_O increase in CSF pressure corresponded to an approximately 13% reduction in SEH odds, confirming low CSF pressure and multilevel decompression as independent predictors.

**Table 3:** Comparison of baseline clinical characteristics between patients with and without postoperative hematoma

Variable	Hematoma group (*n* = 29)	No-hematoma group (*n* = 65)	*P* value
Demographics			
Age, years	75.0 ± 8.5	70.2 ± 10.5	0.039*
Female sex, n (%)	12 (41.4%)	17 (27.3%)	0.154
BMI (kg/m^2^)	24.6 ± 2.6	25.0 ± 3.3	0.594
Medical History			
Smoking, n (%)	5 (17.2%)	15 (22.7%)	0.596
Alcohol use, n (%)	10 (34.5%)	28 (42.4%)	0.377
Diabetes Mellitus, n (%)	3 (10.3%)	9 (13.6%)	0.749
Hypertension, n (%)	16 (55.2%)	41 (63.0%)	0.821
Congestive heart failure, n (%)	0 (0%)	0 (0%)	-
Pulmonary disease, n (%)	0 (0%)	0 (0%)	-
Frailty and functional status			
Functionally dependent, n (%)^a^	3 (10.3%)	4 (6.2%)	0.246
Frail (mFI-5 ≥ 2), n (%)	7 (24.1%)	14 (21.5%)	0.793
ASA-PS ≥ 2, n (%)	2 (6.9%)	9 (13.8%)	0.493
Preoperative measurements			
Pre-operative SBP (mmHg)	130.2 ± 16.1	131.8 ± 15.5	0.644
Pre-operative DBP (mmHg)	76.6 ± 12.1	77.5 ± 10.3	0.696
CSF pressure (mmH_2_O)	127.6 ± 41.2	150 ± 45.1	0.031*
Medication use			
Anticoagulant use, n (%)	2 (6.9%)	6 (9.1%)	0.172
Antiplatelet use, n (%)	0 (0%)	6 (9.1%)	1
Laboratory data			
Platelets (x10^3^/µL)	228.3 ± 55.0	225.6 ± 51.5	0.818
PT-INR	0.98 ± 0.06	0.99 ± 0.06	0.648
APTT (sec)	31.2 ± 5.4	29.5 ± 2.3	0.182

Values are expressed as mean ± SD or number (%)

*BMI* Body mass index, *mFI-5* Modified 5-item frailty index, *ASA-PS* American Society of Anesthesiologists Physical Status, *SBP* Systolic blood pressure, *DBP* Diastolic blood pressure, *CSF* Cerebrospinal fluid, *PT-INR* Prothrombin time–international normalized ratio, *APTT* Activated partial thromboplastin time

^a^Defined as requiring partial or complete assistance with activities of daily living (based on mFI-5)

^*^P < 0.05, considered statistically significant

**Table 4:** Comparison of intraoperative and postoperative variables between groups

Variable	Hematoma group (*n* = 29)	No-hematoma group (*n* = 65)	*P* value
Intraoperative variables			
Operating time (min)	110.1 ± 48.1	108.5 ± 43.6	0.866
Blood loss (mL)	148.2 ± 190.0	113.1 ± 107.1	0.279
Number of levels decompressed	2.6 ± 1.0	2.1 ± 0.9	0.020*
Durotomy, n (%)	2 (6.9%)	3 (4.5%)	0.642
Discectomy, n (%)	11 (37.9%)	18 (27.3%)	0.233
Revision surgery, n (%)	2 (6.9%)	3 (4.5%)	0.404
L2/3 decompression, n (%)	9 (31.0%)	12 (18.2%)	0.190
Postoperative variables			
Total drain output (mL)	331.4 ± 174.8	401.5 ± 227.6	0.151
Maximum SBP (mmHg)	127.1 ± 17.1	132.2 ± 19.7	0.237
Maximum DBP (mmHg)	75 ± 10.7	81.2 ± 11.9	0.022*
Average SBP (mmHg)	128.5 ± 14.8	128.8 ± 16.4	0.940
Average DBP (mmHg)	66 ± 8.7	70.3 ± 12.4	0.095

Values are expressed as mean ± SD or number (%)

*SBP* Systolic blood pressure, *DBP* Diastolic blood pressure

^*^P < 0.05, considered statistically significant

**Table 5:** Multivariable logistic regression analysis for risk factors associated with postoperative SEH

Variable	Adjusted OR (95% CI)	*P* value
CSF pressure (per 10 mmH_2_O)	0.87 (0.74–0.98)	0.028*
Number of levels decompressed	1.94 (1.15–3.47)	0.017*
PT-INR	0.023 (0.000002–128.82)	0.406
Anticoagulant use	2.01 (0.37–16.67)	0.437

*OR* Odds ratio, *CI* Confidence interval, *CSF* Cerebrospinal fluid, *PT-INR* Prothrombin time–international normalized ratio

^*^P < 0.05, considered statistically significant

## Discussion

This study focused on CSF pressure as a potential predisposing factor for postoperative SEH after lumbar decompression surgery. To our knowledge, this is the first clinical study to demonstrate that low preoperative CSF pressure is independently associated with postoperative SEH. Our findings suggest that reduced CSF pressure can contribute to SEH formation, potentially through mechanisms such as epidural venous engorgement and congestion. These results align with prior theoretical and imaging-based hypotheses, but prospective multicenter studies are required to clarify causality and establish whether perioperative CSF pressure modulation can truly reduce SEH risk.

The pathomechanism of postoperative SEH remains unclear. While excessive bleeding from the Batson venous plexus or epidural arteries has been widely accepted as the primary cause [5], these mechanisms alone may not fully account for SEH in patients without identifiable bleeding risk factors. Our findings demonstrated that low CSF pressure is an independent risk factor for SEH, suggesting that pressure dynamics—rather than hemorrhagic tendency alone— plays a significant role in its formation. This perspective provides a potential explanation for previously reported risk factors such as advanced age, female sex, or the use of Gelfoam, which are unrelated to coagulation but influence CSF pressure.

Reduced CSF pressure may facilitate distension of epidural veins and venous congestion, creating a local pressure imbalance that could predispose to minor bleeding and subsequent hematoma accumulation. This mechanism is supported by MRI findings showing epidural venous dilation in patients with CSF hypotension [15, 26], as well as by the nonlinear pressure–volume relationship in the spinal canal, whereby even small increases in epidural volume—such as venous engorgement or minor bleeding—can lead to disproportionate increases in local pressure, especially when CSF pressure is low [27]. Under such conditions, minor venous or capillary disruption may more readily progress to clinically relevant hematoma formation. In addition, epidural venous plexus enlargement under low CSF pressure has been associated with increased mechanical stress on venous walls due to pressure shifts [16]. These observations suggest that maintaining stable CSF dynamics in the early postoperative period may help maintain physiological pressure balance and potentially reduce the risk of SEH, especially in patients with underlying physiological susceptibility.

One practical and non-invasive method to increase lumbar CSF pressure postoperatively is positional adjustment. Current postoperative management typically involves keeping patients in a neutral spine position in the supine or lateral decubitus position. However, CSF pressure is known to vary significantly with body position due to hydrostatic displacement. Animal studies have shown that lumbar CSF pressure increases in proportion to inclination angles, rising by 24% at 30°, 56% at 60°, and 109% at 90° relative to the supine position [28]. Consistent with these findings, clinical data demonstrated a 36% increase in lumbar CSF pressure at a 20° head elevation and 64% at 40° [29]. In the upright position, lumbar CSF pressure can exceed 400 mmH_2_O compared with 50–150 mmH_2_O in the supine position [30]. These findings suggest that maintaining a low Fowler’s position (mild head elevation), as tolerated postoperatively, may help stabilize CSF dynamics and reduce epidural venous congestion, thereby minimizing the risk of SEH. However, such interventions must be cautiously applied in cases involving incidental durotomy or intradural procedures. Low Fowler’s position and early mobilization increase lumbar CSF pressure but decrease intracranial pressure. In such cases, high lumbar CSF pressure can facilitate CSF leakage, whereas low intracranial pressure may increase the risk of intracranial complications, such as subarachnoid hemorrhage, subdural hematoma, and postural headaches. Nevertheless, maintaining mild upper-body elevation together with adequate hydration appears physiologically reasonable and may help reduce epidural venous congestion and postoperative hematoma formation.

Another well-known risk factor for postoperative SEH is intraoperative dural injury [6, 7]. Dural tears can lead to CSF leakage, resulting in a reduction of CSF pressure. Lumbar puncture studies have demonstrated that removal of just 1 mL of CSF can decrease opening pressure by approximately 10 mmH_2_O [31, 32]. Ongoing CSF leakage following durotomy may further reduce CSF pressure, potentially contributing to the development of postoperative SEH [28]. This association between durotomy and increased SEH risk highlights the importance of achieving a tight, watertight dural closure, which helps restore normal CSF dynamics and reduce postoperative complications [33].

Dehydration also contributes to reduced CSF pressure by impairing CSF production through decreased cerebral blood flow [34]. Given the exponential relationship between CSF volume and pressure, adequate perioperative hydration is essential for maintaining stable CSF dynamics and may represent a practical strategy to reduce the risk of SEH.

Massive intraoperative blood loss has been identified as a risk factor for SEH [5, 24]. Significant bleeding may reduce CSF pressure by impairing cerebral blood flow secondary to hypovolemia. Furthermore, severe hemorrhage can induce coagulopathy, which may in turn increase the risk of hematoma formation [2, 6, 24, 25]. In such cases, timely transfusion of blood products—including fresh frozen plasma—is critical for restoring circulatory volume, correcting coagulopathy, and preserving both hemodynamic and CSF homeostasis.

Furthermore, patient-specific factors such as age and sex are established determinants of CSF pressure [22, 35]. Women and older patients tend to have lower CSF pressure. Advancing age is associated with reduced CSF production and intracranial compliance, leading to lower CSF pressure. Similarly, women tend to have lower baseline CSF pressure than men, likely due to anatomical and hormonal differences. The higher incidence of postoperative SEH reported in elderly female patients [25] may thus, in part, be attributable to lower baseline CSF pressure, reinforcing the clinical relevance of our findings and the need for tailored perioperative management. In addition, previous studies have reported a positive association between BMI and CSF pressure [36], suggesting that body habitus may also influence CSF dynamics. However, in the present cohort, BMI itself was not significantly associated with postoperative SEH, indicating that the relationship between BMI, CSF pressure, and SEH is likely complex and not mediated through a single pathway. Although preoperative lumbar puncture is not routinely performed in all clinical settings, CSF pressure may serve as a physiological indicator of systemic volume status and venous congestion, offering potential guidance for perioperative fluid management. Even in the absence of direct measurement, maintaining stable CSF dynamics through appropriate perioperative positioning and hydration may help reduce the risk of postoperative SEH.

This study has several limitations. First, its retrospective, single-institution design and relatively limited sample size may introduce selection bias and limit generalizability. Second, the timing of postoperative MRI was not standardized, and CSF pressure is posture-dependent; therefore, measurement-related variability should be considered when interpreting the results, including factors related to postoperative drain management, although preoperative CSF pressure was analyzed as a baseline physiological characteristic. Third, because the number of patients undergoing laminectomy was very small, potential differences between laminotomy and laminectomy could not be evaluated. Fourth, patients undergoing fusion surgery were excluded; therefore, potential differences in perioperative CSF dynamics and hemostatic strategies between fusion and decompression-alone procedures could not be evaluated. Fifth, clinical neurological outcomes were not analyzed due to the low number of symptomatic cases. Therefore, further prospective multicenter studies with standardized imaging protocols would be valuable to confirm and extend these findings.

## Conclusions

This study identified low CSF pressure as an independent risk factor for postoperative SEH and a potentially modifiable physiological factor. Perioperative strategies aimed at maintaining adequate CSF pressure may help reduce the risk of epidural hematoma, pending confirmation in prospective studies.

## Data Availability

The datasets generated and analyzed during the current study are available from the corresponding author on reasonable request.
